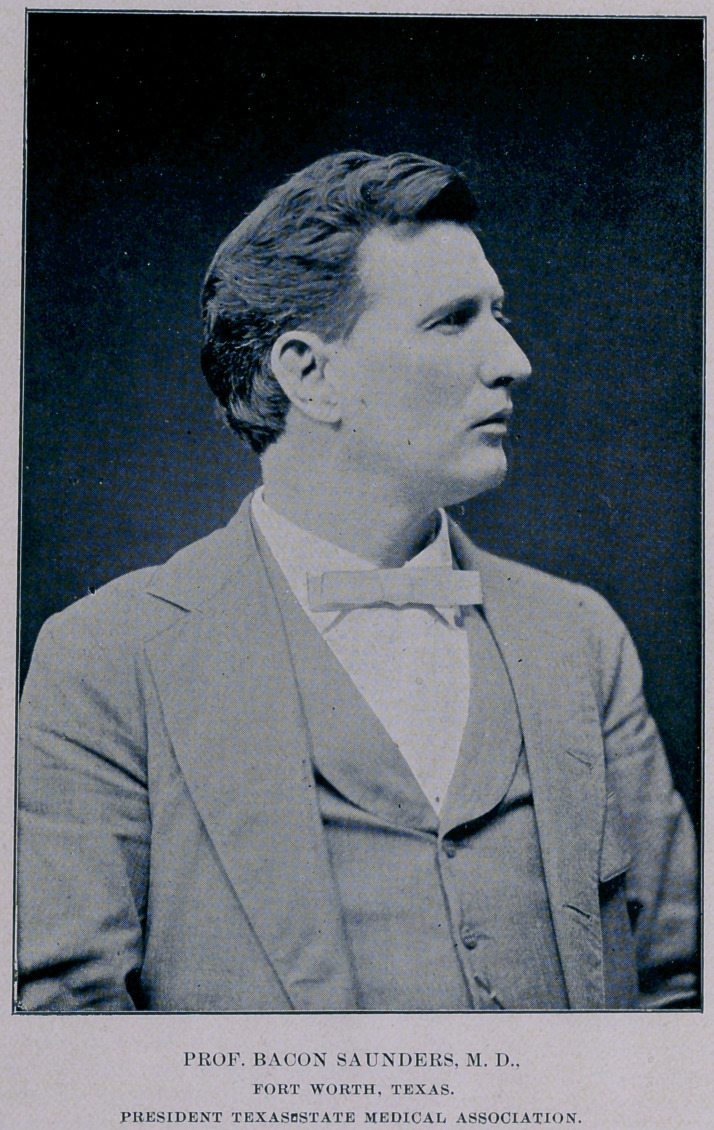# Biographical—Prof. Bacon Saunders, M. D., President Texas State Medical Associations

**Published:** 1897-06

**Authors:** 


PROF. BACON SAUNDERS, M. D.,

FORT WORTH, TEXAS.

PRESIDENT TEXAS STATE MEDICAL ASSOCIATION.

### Biographical.

For the Texas Medical Journal.

### Prof. Bacon Saunders, M. D., President Texas State Medical Association.

This gentleman is the son of Dr. John S. Saunders, deceased, and Mrs. Sarah J. Saunders (nee Claypool), who yet survives. He was born on January 5, 1855, near Bowling Green, in Warren county, Ky., the good old State, which from its early population of hardy and brave pioneers has, in this instance as in many others, been beneficient in contributing to the worthy citizenship of its younger sister States.

With his parents, Dr Saunders moved from Kentucky in the fall of 1857, to Dallas, Texas, where the family resided until October, 1869, when they removed to Bonham, in Fannin county. Here the subject of this sketch was educated in the literary department of Carlton College. Thereafter, in 1873, he began the study of medicine in his father's office, whence he entered the medical department of the University of Louisville, Ky., in September, 1875. From that institution he graduated in March, 1877, receiving the gold medal for the highest honors of his class, and taking a special prize for proficiency in operative surgery. Returning to Bonham, he entered into partnership with his father, in the practice of medicine. On October

31, 1877, he was married to Miss Ida Caldwell, of Bonham, a charming, cultured and most estimable lady. At the latter place he practiced his profession with diligence and success until January, 1893, when, with his family, consisting of his wife and two children, Roy Farra and Linda Ray, he moved to Fort Worth, becoming a member of the firm of Adams, Thompson & Saunders.

Dr. Saunders is a member of the Americal Medical Association, the Southern Surgical and Gynecological Association, and the American Academy of Railway Surgeons. He is a member and ex-president of the North Texas Medical Association, and a member and one of the organizers of the Northwest Texas Medical Association. He is an honorary member of the Grayson Medical Society, one of the organizers of the medical department of the Fort Worth University, in which institution he occupies the chair of Operative and Clinical Surgery, and is also dean of the faculty. He is assistant chief surgeon of the Texas & Pacific, the Santa Fe, the Chicago, Rock Island & Texas, and the St. Louis Southwestern railway companies. He is the Medical Referee for Texas of the Mutual Life Insurance Company of New York.

While enjoying a large and lucrative practice in the successful treatment of diseases, Dr. Saunders yet makes the field of surgery the subject of special cultivation. In the practice of this noble science, and in the performance of numerous and successful operations, he has evinced remarkable skill.

The greatest prize for virtue and merit which humanity can bestow is the esteem and honor of one's fellow citizens and professional brethren. This prize has been won by Dr. Saunders, and that he will live many years to wear it worthily is at once the wish and the prophecy of A LAY FRIEND.

## Figures and Tables

**Figure f1:**